# Capsaicin, a Phytochemical From Chili Pepper, Alleviates the Ultraviolet Irradiation-Induced Decline of Collagen in Dermal Fibroblast *via* Blocking the Generation of Reactive Oxygen Species

**DOI:** 10.3389/fphar.2022.872912

**Published:** 2022-03-14

**Authors:** Qiyun Wu, Panzhu Bai, Hongsheng Guo, Maggie S. S. Guo, Yingjie Xia, Yiteng Xia, Xiong Gao, Xiaoyang Wang, Jiahui Wu, Tina T. X. Dong, Karl W. K. Tsim

**Affiliations:** ^1^ Division of Life Science and Center for Chinese Medicine, The Hong Kong University of Science and Technology, Hong Kong, Hong Kong SAR, China; ^2^ Shenzhen Key Laboratory of Edible and Medicinal Bioresources, HKUST Shenzhen Research Institute, Shenzhen, China

**Keywords:** capsaicin, collagen, fibroblast, ERK, reactive oxygen species

## Abstract

Capsaicin, a major ingredient in chili pepper, has broad pharmaceutical applications, including relieving pain, anti-inflammation, and treating psoriasis. In dermatological biology, capsaicin has been shown to prevent the ultraviolet (UV)-induced melanogenesis *via* TRPV1 receptor. To strengthen the roles of capsaicin in skin function, the damaged skin, triggered by exposure to UV, was reversed by capsaicin in both *in vitro* and *in vivo* models. In cultured dermal fibroblasts, the exposure to UV induced a decrease of collagen synthesis and increases expression of matrix metalloproteinases (MMPs), generation of reactive oxygen species (ROS), and phosphorylation of Erk and c-Jun, and these events subsequently led to skin damage. However, the UV-mediated damages could be reversed by pre-treatment with capsaicin in a dose-dependent manner. The effect of capsaicin in blocking the UV-mediated collagen synthesis was mediated by reducing generation of ROS in dermal fibroblasts, instead of the receptor for capsaicin. Hence, capsaicin has high potential value in applying as an agent for anti-skin aging in dermatology.

## Introduction

Skin aging is a complex biological phenomenon having two specific causes: 1) inherent aging triggered by mostly genetic factors; and 2) external aging triggered by exposure to environmental stimuli (Jenkins, 2002). Photo-aging is the unique clinical, histological, and functional characteristics of skin being exposed to sun for a long time (Wlaschek et al., 2001). There are distinct characteristics in photo-aged skin with an independent pathological phenomenon, which distinguish it from naturally aging skin (Scharffetter–Kochanek et al., 2000). The inherent aging process of skin is similar to that of most internal organs, showing a decline in proliferating ability of cells, and thereafter leads to decrease of cellular metabolic activity and cell death (Kohl et al., 2011). Photo-aging results in changes in capacity of cell biosynthesis and a disorder of dermal matrix (Berneburg et al., 2000). The clinical manifestations are leathery appearance, increased wrinkle formation, reduced recoil capacity, increased fragility of skin with blister formation, and impaired wound healing (Wlaschek et al., 2001; Jenkins, 2002). Due to the demand from clinical and cosmetic industry, this is necessary to explore the agents that prevent photo-aging of skin.

Skin is the primary target of ultraviolet (UV) irradiation. As the most abundant component of human connective tissue, collagen is a structural protein in skin (Shoulders and Raines, 2011). Collagen composes around 33% of total protein, even up to 75% of the dry weight, of skin in human body (Cheng et al., 2011), and it is the most common component of extracellular matrix. The structure of native collagen is a triple-helix conformation, i.e., three polyproline II helical supercoil with each other and having a common one-residue axis, as to stabilize its structure (Shoulders and Raines, 2011). In human skin, type I and type III collagens (COL1 and COL3) are the dominant subtypes. COL1 is a heterotrimer having two α1 and one α2 chains (Jariashvili et al., 2012), which is always threatened by UV-induced damage (Shoulders and Raines, 2011; Jariashvili et al., 2012). Several lines of evidence have suggested that UV exposure could induce oxidative degradation of collagen, and consequently expedites the skin damage during aging (Tanaka et al., 2009; Jariashvili et al., 2012). One of the assumptions of collagen degradation is the disruption of peptide bonds within collagen under UV exposure, rather than damaging the helical structure (Kamińska and Sionkowska, 1996).

Capsaicin is a major component of chili peppers, about 8% of total dried weight, and its secondary metabolites are capsaicinoids (Huang et al., 2000). Capsaicin is irritating to mammals and produces burning sensation in the tissues (Cordell and Araujo, 1993). Capsaicin is an agonist of transient receptor potential cation channel subfamily V member 1 (TRPV1) in skin, leading to an irritating mechanism (Fitzgerald, 1983). After binding to TRPV1 on the membrane, capsaicin activates the ion channel directly coupled to this receptor, inducing Ca^2+^ entering the cell (Rosenbaum et al., 2004). This action depolarizes the painful neurons and triggers an action potential, and eventually produces a sense of spiciness (Caterina et al., 1997). In contrast, capsazepine, a competitive antagonist for TRPV1, can reversibly reduce, or abrogate, the response to capsaicin (Bevan et al., 1992).

Beside the application in food additives, capsaicin has been reported to have functions in medical usage, such as relieving pain, anti-inflammation, and treating psoriasis (Lam and Ferrell, 1989; Winter et al., 1995; Agrawal et al., 2015). Our previous work reported that capsaicin inhibited the synthesis of melanin in murine melanocytes, as further illustrated *in vivo* and *in vitro* model (Wu et al., 2020). Here, we revealed the function of capsaicin in preventing the photo-aging of skin. In cultured dermal fibroblasts, application of capsaicin at low dose alleviated UV-induced decline of collagen. The cell-based experiments verified that capsaicin promoted collagen synthesis in dermal fibroblasts *via* reactive oxygen species (ROS)-mediated extracellular signal-regulated kinases (Erk) and/or c-Jun signaling. These findings suggest that capsaicin, as a topical medicine, has a strong potential as a cosmetics ingredient in application for anti-photoaging.

## Materials and Methods

### Chemicals

Capsaicin, capsazepine, transforming growth factor beta 1 (TGF-β1), N-acetyl-l-cysteine (NAC), 2′-7′ dichlorofluorescin diacetate (DCFH-DA) and PD98058 were all purchased from Sigma-Aldrich (St Louis, MO). PAF C-16 was purchased from Santa Cruz Biotechnology (Dallas, TX).

### Cultures of Dermal Fibroblasts and Keratinocytes

Murine dermal fibroblasts were isolated from C57BL/6 mice, as described previously (Seluanov et al., 2010). Briefly, the dorsal skin was harvested from day 2 mouse, after sacrificed. The collected skin was incubated at 4 mg/ml dispase overnight at 4°C. Afterwards, the dermis was separated from the epidermis and then digested in 3 mg/ml collagenase for 1 h at 37°C. After filtration through 40 µm cell strainer, the dermal fibroblasts were harvested by centrifugation at 180 × g for 3 min. Cells were cultured in Dulbecco’s modified eagle medium (DMEM) with 20% fetal bovine serum (FBS). Primary murine epidermal keratinocytes were obtained, as described previously (Li et al., 2017) and cultured in EpiLife™ medium with 60 μM calcium. The medium was supplemented with 1% (v/v) penicillin/streptomycin (10,000 U and 10,000 μg/ml), and the cells were incubated in a humidified atmosphere with 5% CO^2^ at 37°C. The culture reagents were purchased from Thermo Fisher Scientific (Waltham, MA).

### Cell Viability Assay

MTT [3-(4,5-dimethylthiazolyl-2)-2,5-diphenyltetrazolium bromide] assay was performed to determine cell viability with drug treatments. Cells were seeded onto 96-well plates for 24 h and treated with various concentrations of capsaicin for further 24 h. Afterwards, the medium was removed, and 100 μl of MTT (0.5 mg/ml in complete growth medium) solution was added and incubated at 37°C for 2 h. The medium was replaced with 100 μl of DMSO in each well and shaken for 15 min. Absorbance was measured using a Multiskan FC Microplate Photometer (Thermo Fisher Scientific) at a wavelength of 570 nm.

### Animal Study

Animals were obtained from Animal and Plant Care Facility of Hong Kong University of Science and Technology (HKUST), and the experiments were performed according to the guidelines of Department of Health, The Government of Hong Kong SAR. The experimental procedures were approved by Animal Ethics Committee at the University (Reference No (15–50) in DH/SHS/8/2/2 Pt.2). The female C57BL/6 mice at 8 weeks old were divided into four groups. The hair on dorsal skin was shaved. The mice in ultraviolet B (UVB) and capsaicin + UVB groups were pre-treated with saline and capsaicin, respectively, on dorsal skin each day, followed by exposed to UVB irradiation (300 mW/m^2^ for 30 min each day) generated by Philips UVB narrowband lamp (Philips, Amsterdam, Netherlands) for 5 days. The control and capsaicin group were treated with saline and capsaicin, respectively, without exposure to UVB irradiation. The dorsal skins were collected after the mice were sacrificed by cervical dislocation, for haematoxylin and eosin staining, and immuno-histochemistry.

### *Ex Vivo* Mouse Skin

The dorsal skins were excised from C57BL/6 mice (8 weeks old) after sacrificed by cervical dislocation with the hair shaved, and then maintained in DMEM supplemented with 10% (v/v) FBS and 1% (v/v) penicillin/streptomycin (10,000 U and 10,000 g/ml) in 5% CO_2_ at 37°C. The skin was treated with drugs followed by exposure to UVB irradiation (300 mW/m^2^ for 30 min each day) for 3 days. The protein and total RNA were extracted from the *ex vivo* skins.

### qRT-PCR

Total RNA was extracted using RNAzol^@^RT reagent (Molecular Research Center, Cincinnati, OH). Briefly, the homogenized mouse skins, or cells, were incubated in RNAzol^@^RT at room temperature. Then, total RNA was precipitated in 75% ethanol (v/v) by centrifugation at 12,000 × g for 10 min. The RNA pellet was washed by 75% ethanol and dissolved in RNAase-free water. The RNA quality was determined according to a ratio (∼2.0) of absorbance at 260–280 nm by NanoDrop™ (Thermo Fisher Scientific). One μg RNA of each sample was applied for reverse transcription using First Strand cDNA Synthesis Kit (Thermo Fisher Scientific), in accord with the manufacturer’s protocol. The sequences of specific primers were shown in Table 1. Amplification was performed for 45 cycles. Each cycle consisted of denaturation at 95°C for 30 s, annealing at 55°C for 30 s, and extension at 72°C for 20 s, performed on Roche Lightcycler 480 System (Roche, Basel, Switzerland).

**TABLE 1:** Primer sequences for relative qRT-PCR analysis.

Gene	Species	Forward sequence (5′–3′)	Reverse sequence (5′–3′)
COL1A1	mouse	GAG​CGG​AGA​GTA​CTG​GAT​CG	GCT​TCT​TTT​CCT​TGG​GGT​TC
COL1A2	mouse	CCA​GCG​AAG​AAC​TCA​TAC​AGC	GGA​CAC​CCC​TTC​TAC​GTT​GT
COL3A1	mouse	TCC​TGG​TGG​TCC​TGG​TAC​TG	AGG​AGA​ACC​ACT​GTT​GCC​TG
MMP1a	mouse	GCC​AGA​ACC​TGA​GCT​CAA​TTT​AAT​A	GCC​CAT​ACT​TTG​CTG​CCT​TT
MMP3	mouse	CCC​TGG​GAC​TCT​ACC​ACT​CA	GCT​GTG​GGA​GTT​CCA​TAG​AGG
MMP9	mouse	CTC​TCC​TGG​CTT​TCG​GCT​G	AGC​GGT​ACA​AGT​ATG​CCT​CTG
GAPDH	mouse	AAG​GTC​ATC​CCA​GAG​CTG​AA	CTG​CTT​CAC​CAC​CTT​CTT​GA

### Haematoxylin and Eosin Staining

The dorsal skin was collected from mice after sacrificed. Skin tissues were embedded in optimal cutting temperature (OCT) compound by snap freezing in liquid nitrogen and then stored at −80°C for further experiments. The skin section at 10 µm-thickness was made using Thermo CryoStar X 70 Cryostat (Thermo Fisher Scientific), followed by fixation with 4% paraformaldehyde for 10 min at room temperature. After sectioning, skin section was subjected to haematoxylin and eosin staining, according to the protocol of a staining kit (Yuan Ye Biology, Shanghai, China) with minor modification. Briefly, skin section was incubated in haematoxylin solution for 5 min followed by 1% (v/v) acid alcohol. Then, the skin section was incubated with eosin for 3 s before dehydration with 95 and 100% ethanol, then incubated with xylene for 15 s for three times.

### Immunohistochemistry

The pre-fixed skin sections were incubated with 30% hydrogen peroxide for 10 min and then blocked at room temperature for 1 h. Afterwards, skin sections were incubated with primary antibody anti-collagen type I (Abcam Ltd., Cambridge, UK) at 4°C overnight then with horseradish peroxidase (HRP)-conjugated secondary antibody (Sigma-Aldrich) at room temperature for 2 h. The signal was detected using DAB substrate kit (Abcam Ltd.) and further stained with haematoxylin for the nuclear followed by mounted.

### Immunofluorescent Staining

The cultured cells were fixed with 4% paraformaldehyde for 10 min. Samples were incubated with 1% BSA with 0.2% Triton X-100 for 1 h. Cultures were incubated with primary antibodies, anti-cytokeratin at 1:100 (Abcam Ltd.), anti-collagen type I at 1:100 (Abcam Ltd.), at 4°C overnight, followed by incubation with Alexa 488/647-conjugated antibodies (Abcam Ltd.). Samples were mounted with ProLong™ Gold Antifade Mountant with DAPI (Thermo Fisher Scientific) and then subjected to visualised by Zeiss laser scanning confocal microscope.

### Measurement of Total Collagen

The amounts of collagen in mouse skin and cultured dermal fibroblasts were measured using a collagen ELISA kit (Shanghai Fankel Industrial Co., Shanghai, China), according to the protocol. Briefly, after exposure to UVB or treatment with various drugs, the protein lysates were extracted from mice dorsal skin and cultured fibroblasts, and incubated with reaction reagents at room temperature for 1 h. The amount of collagen was determined by the absorbance at 450 nm according to the standard curve of human collagen.

### SDS-PAGE and Western Blot Analysis

The protein lysates were extracted from murine dermal fibroblasts with RIPA lysis buffer, and 40 μg of each was subjected to 8% sodium dodecyl sulfate-polyacrylamide gel electrophoresis (SDS-PAGE) transferred to nitrocellulose membranes. The membranes were blocked with 5% skim milk powder in Tris-buffered saline with 0.1% Tween-20 (TBST) for 2 h. After blocking, the membranes were incubated at 4°C overnight with specific primary antibodies, including anti-collagen type I at 1:1,000 (Abcam Ltd.), anti-Erk 1/2 (Cell Signaling Technology, Danvers, MA) at 1:1,000, anti-phospho-Erk 1/2 (Cell Signaling Technology), at 1:1,000, and anti-α tubulin at 1:2,000 (Sigma-Aldrich), followed by incubated with HRP secondary antibodies at room temperature for 1 h. The immune-reactive proteins were detected using enhanced chemiluminescence (ECL) western blotting detection kit (Thermo Fisher Scientific). The intensities of bands were quantified using ChemiDoc Imaging System (Bio-Rad).

### Measurement of Intracellular Ca^2+^

Mouse dermal fibroblasts were loaded with calcium indicator by incubation with 2 μM fluo-4 AM (Thermo Fisher Scientific) in culture medium at 37°C for 30 min. Then, the cells were observed with drug treatment and the image was captured by Zeiss confocal microscope. The intensity of fluorescent signal was quantified using ImageJ software (NIH Image).

### Measurement of Intracellular ROS

The cultured dermal fibroblasts were pre-treated with capsaicin for 30 min, followed by exposure to UVB irradiation at 300 mW/m^2^ for 5 min. After 6 h, cells were washed with PBS twice and then incubated with DCFH-DA (20 µM) in serum-free medium at 37°C for 20 min. For image captured by confocal microscope, cells were fixed with 4% paraformaldehyde for 10 min at room temperature and then mounted with ProLong™ Gold Antifade Mountant with DAPI (Thermo Fisher Scientific) followed by visualizing through a Zeiss laser scanning confocal microscope. The intensities of the fluorescent signals were quantified using ImageJ software.

### DNA Transfection and Luciferase Assay

The DNA construct pARE-Luc containing four antioxidant response elements (ARE; 5′-TGA nnn GCA-3′) and a downstream luciferase-reporter gene was used. Cultured fibroblasts were transfected using jetPRIME reagent (Polyplus Transfection, New York, NY), as described (Lai et al., 2021). The pARE-Luc transfected cells were pre-treated with capsaicin for 30 min, followed by exposure to UVB irradiation. The promoter-driven luciferase assay was performed using a commercial kit (Thermo Fisher Scientific). In brief, cells were lysed by 100 mM potassium phosphate buffer (pH 7.8), 0.2% Triton X-100 and 1 mM DTT and agitated for 30 min. Afterwards, cells were centrifuged at 16,100 × g for 10 min at 4°C. Twenty micrograms of cell lysates were used for assay. The luminescent reaction was quantified in a GloMax^®^ 96 Microplate Luminometer and normalized by protein amount.

### Statistical Analysis

Data are expressed as the mean ± SD. Statistical comparisons of the means between different treatments were analysed by using one-way ANOVA followed by a Bonferroni post-hoc test. Significant values are presented as *p < 0.05, **p < 0.01, and ***p < 0.001.

## Results

### Capsaicin Alleviates the UV-Induced Reduction of Collagen in Mouse Skin

**FIGURE 1:**
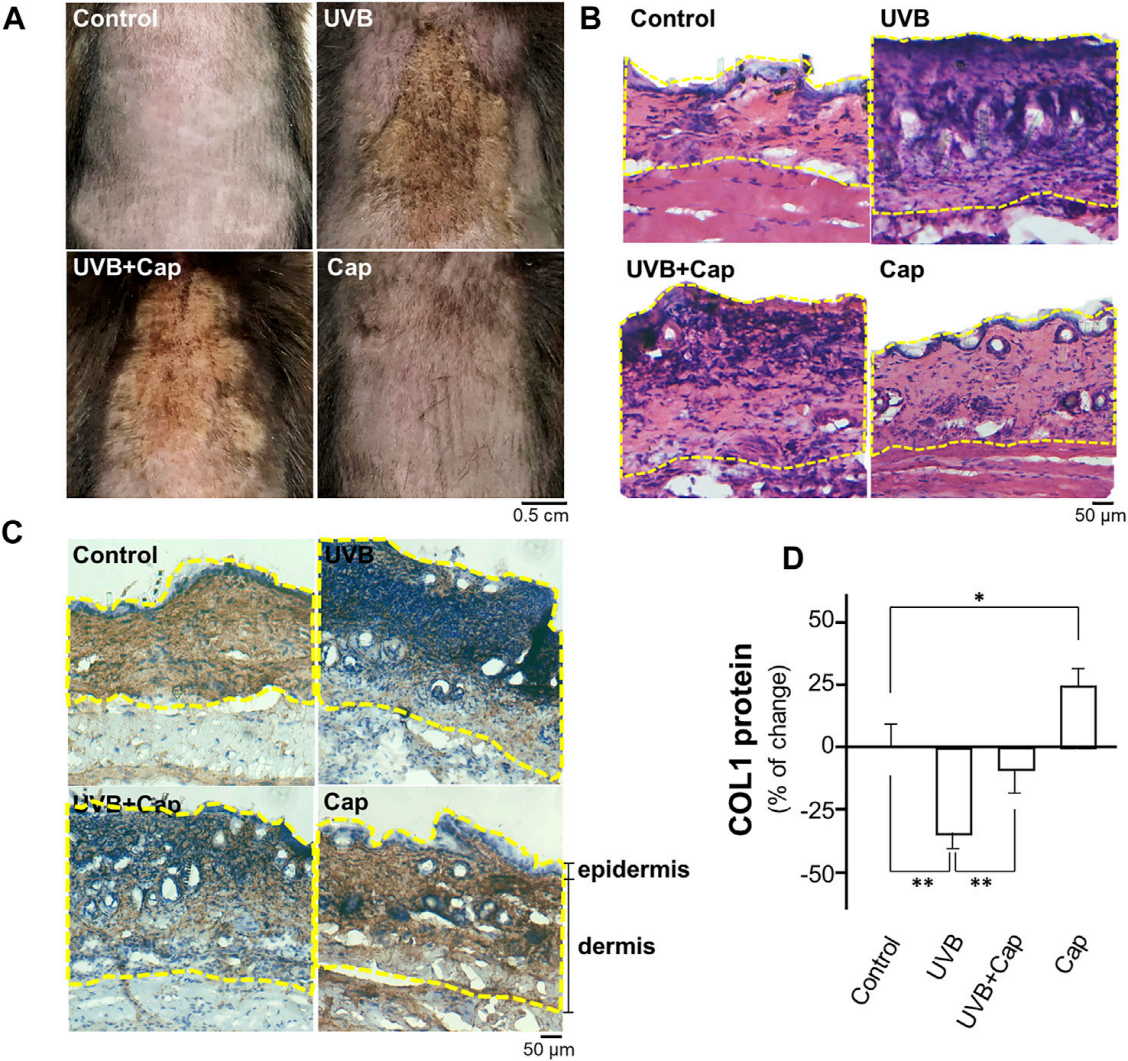
Capsaicin alleviates the UV-induced reduction of collagen in mouse skin *in vivo*. Female C57BL/6 mice, 8 weeks old, were shaved at dorsal skin and divided into four groups: Control (vehicle only); UVB (exposed to UVB irradiation at 300 mW/m^2^ for 30 min each day); UVB + Cap (pre-treated with capsaicin at 5 μM and exposed to UVB irradiation at 300 mW/m^2^ for 30 min each day); Cap (treated with capsaicin at 5 μM each day). **(A)** Mice were sacrificed, and the photos of dorsal skin were taken. **(B)** The 10-μm skin section was stained with H&E staining to show the nucleic acid (purple), cytoplasm and extracellular matrix (red). **(C)** IHC staining was performed to show the expression and localization of COL1 (collagen type I; brown) and nucleic acid (blue) in skin. Dotted line marked the epidermis and dermis layers. **(D)** The protein lysates of dorsal skin were collected for ELISA assay to measure COL1 content. Values are in percentage of change against control, in mean ± SD, *n* = 5, each with triplicate samples. *, *p* < 0.05; **, *p* < 0.01 compared with control (vehicle only).

The shaved mouse dorsal skins were topically treated with, or without, capsaicin for 30 min before stimulated by UVB irradiation (300 mW/m^2^ for 30 min) every day. After 5 days of irradiation, the skin condition was assessed by staining with haematoxylin and eosin, as well as immunostaining using antibody against collagen type I. In the UVB-irradiated group, the skin was drier and showed significant pachylosis, compared with that of control group (no UVB) (Figure 1A). The dryness and pachylosis of skin were considerably alleviated by pre-treatment with capsaicin before the irradiation. Capsaicin itself did not affect the skin condition (Figure 1A). The skin histological result illustrated that the inflammatory responses, e.g., increased thickness and nuclear staining of skin, were triggered markedly under UVB exposure. However, the damages were significantly relieved by applied capsaicin (Figures 1A,B). Furthermore, capsaicin promoted collagen type I (COL1) content in the UVB-irradiated skin back to the control level, as shown by the staining of collagen (Figure 1C) and ELISA assay (Figure 1D). Capsaicin alone increased the level of collagen type I of unexposed skin by ∼25%. These results therefore implied that capsaicin was able to protect the skin from UVB irradiation, especially by promoting the amount of collagen.

**FIGURE 2:**
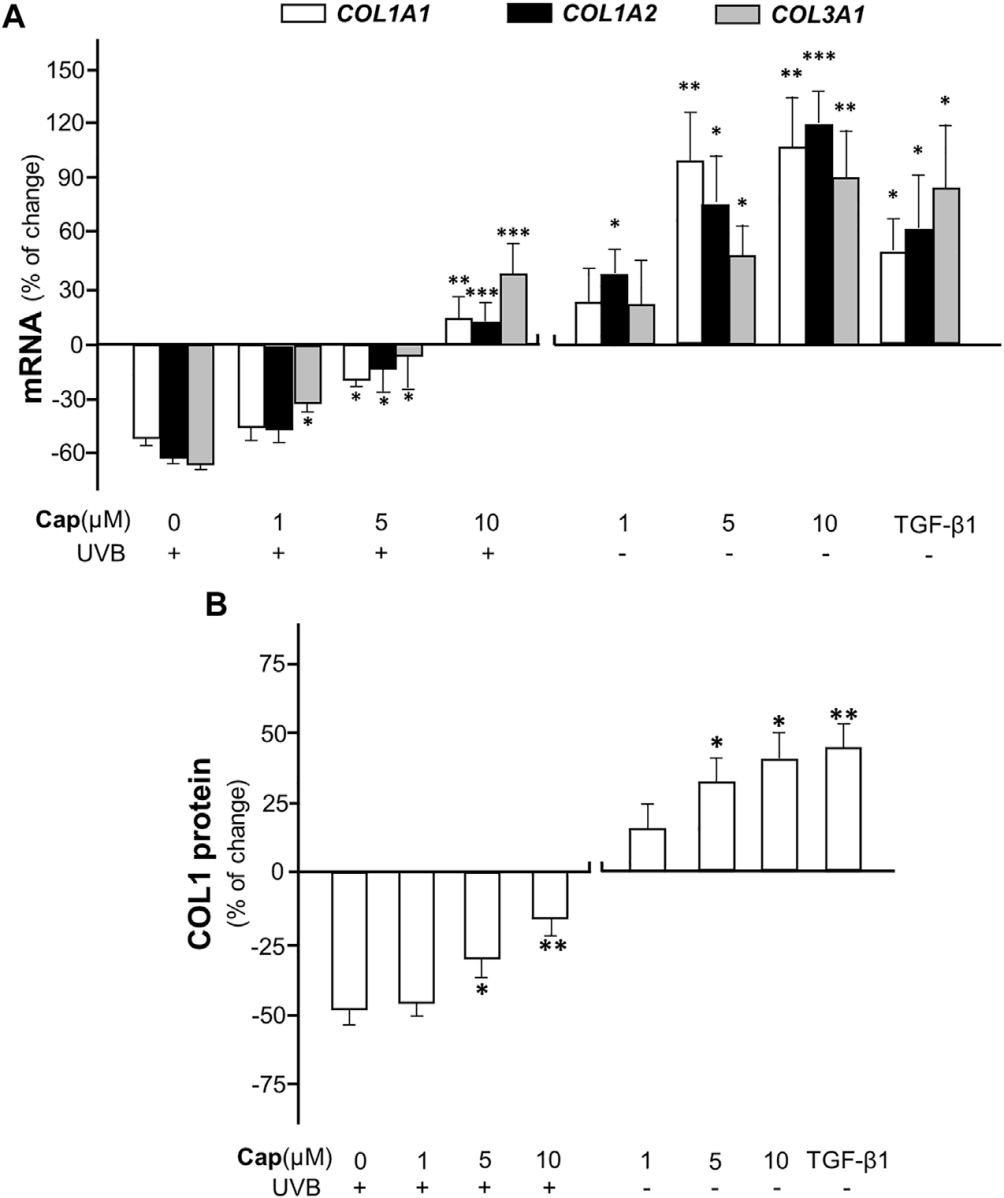
Capsaicin promotes collagen synthesis in dorsal skin *ex vivo*. The shaved dorsal skin was isolated from mice after sacrificed and incubated in medium with capsaicin (Cap) from 1 to 10 μM with or without UVB irradiation at 300 mW/m^2^ for 30 min each day. **(A)** The RNA was extracted for RT-qPCR to relatively determine the transcription of COL1A1, COL1A2, and COL3A1. **(B)** The protein was extracted for ELISA assay to determine the expression of COL1 protein. TGF-β1 (5 ng/ml) was used as a positive control here. Values are in percentage of change against control, in mean ± SD, *n* = 5, each with triplicate samples. *, *p* < 0.05; **, *p* < 0.01; ***, *p* < 0.001 compared with control (vehicle only).

To confirm the effect of capsaicin on collagen production, various concentrations (1–10 μM) of capsaicin were applied onto an *ex vivo* mouse skin culture model. The isolated skin was treated with applied capsaicin. The mRNA levels of different forms of collagen, i.e., COL1A1, COL1A2, and COL3A1, were determined. The result showed that UVB irradiation reduced the transcriptional levels of these genes by ∼60%, dramatically, as compared to the control (no UVB). However, the pre-treatment of capsaicin reversed the UVB-induced decline on the mRNA levels of COL1A1, COL1A2, and COL3A1, indicating a dose-dependent effect in promoting the gene transcriptions (Figure 2A). The maximal concentration of capsaicin used here was 10 μM, which restored the mRNA levels to over 20% of increase, as compared to the control. On the other hand, the treatment of capsaicin only promoted the mRNA levels of these genes in the non-UVB exposed mouse skins, again in a dose-dependent manner (Figure 2A). The mRNA expression of collagen could be maximized at ∼100% increase of the control under the challenge of 10 μM capsaicin. In parallel, the protein level of COL1 showed similar tendency, in which capsaicin induced collagen content in skin with or without UVB irradiation (Figure 2B). This inductive effect of capsaicin was in a dose-dependent manner as well. TGF-β1, a well-known promoter on collagen synthesis in fibroblast, was applied as a positive control.

### Capsaicin Induces Collagen Synthesis *via* Erk-Mediated MMP Signaling

In order to reveal the roles of capsaicin in skin cell models, two cell types, keratinocytes and dermal fibroblasts, were isolated from mouse skin and cultured *in vitro*. The cell identities were validated by morphology and antibodies against collagen type I for fibroblast, or cytokeratin for keratinocyte, in immunofluorescent staining (Supplementary Figure S1A). By MTT assays, the toxicity dose of capsaicin in cell viabilities of both fibroblasts and keratinocytes was determined: the concentration from 1 to 10 μM showed no cytotoxicity to the cultures (Supplementary Figure S1B). Thus, capsaicin did not show toxicity at our working concentrations to the major cell types of skin.

**FIGURE 3:**
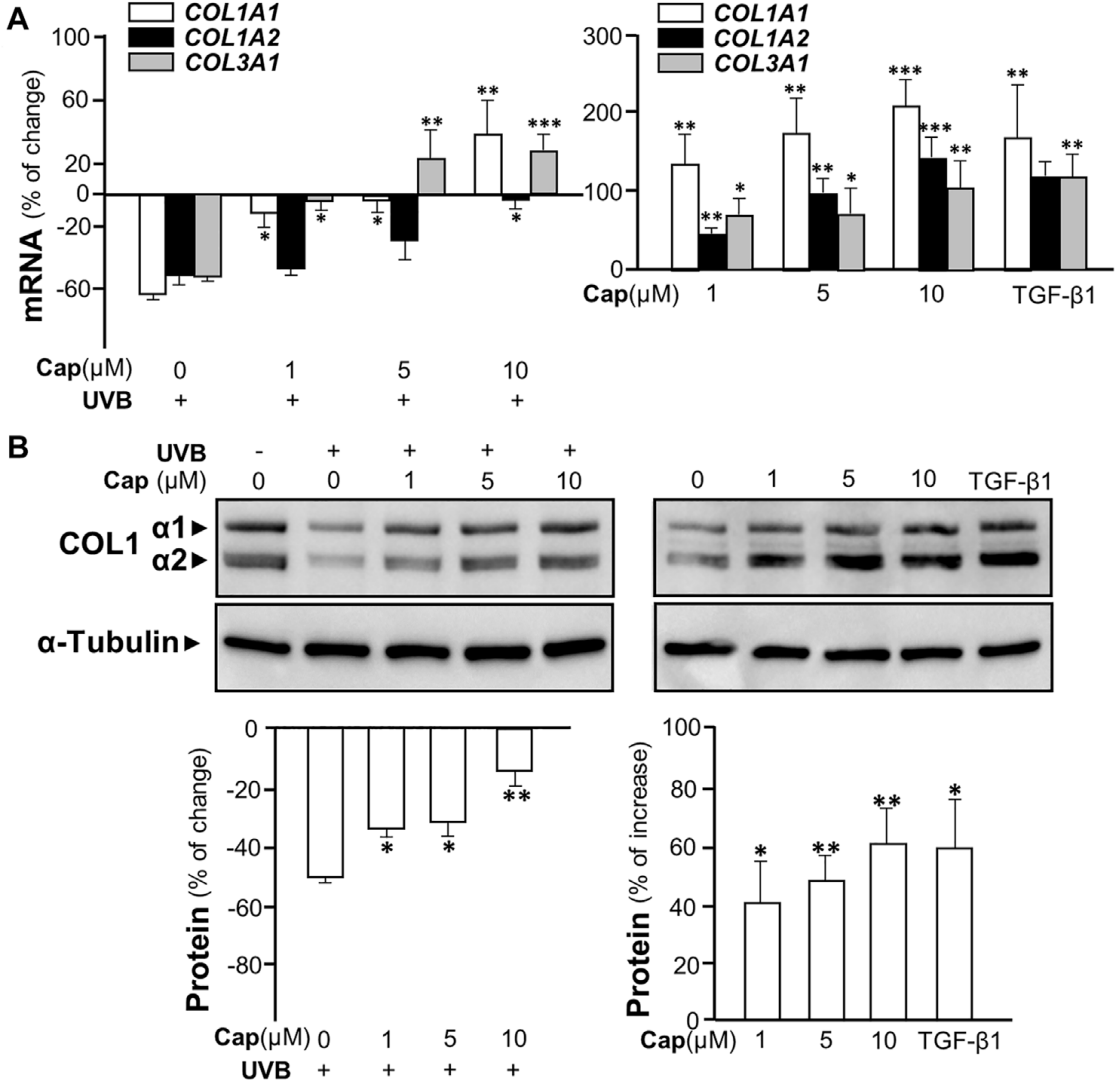
Capsaicin alleviates UVB-induced decrease on collagen in cultured fibroblasts. The dermal fibroblasts were isolated from postnatal day 2 mice and cultured *in vitro*. Cells were incubated with capsaicin (Cap) from 1 to 10 μM with or without UVB irradiation at 300 mW/m^2^ for 5 min. **(A)** The mRNAs of COL1A1, COL1A2, and COL3A1 were determined using qRT-PCR with (left panel) or without (right panel) UVB. **(B)** The protein lysates were collected for SDS-PAGE and Western blot using antibody against COL1α1 (∼138 kDa) and α2 (∼129 kDa) with (left panel) or without (right panel) UVB. Antibody against α-tubulin (∼55 kDa) was used as a loading control. TGF-β1 (5 ng/ml) was used as a positive control here. The quantification of the blots was shown below (bottom panels). Values are in percentage of change, or increase, against control, in mean ± SD, *n* = 5, each with triplicate samples. *, *p* < 0.05; **, *p* < 0.01; ***, *p* < 0.001 compared with control (vehicle only).

The irradiation of UVB suppressed the transcriptional levels of COL1A1, COL1A2, and COL3A1 in cultured fibroblasts by over 50%, similar to that in animal tests (Figure 3A, left panel). The mRNA levels of these genes were increased significantly by pre-treatment of capsaicin in cultured fibroblasts being exposed to UVB. Meanwhile, the treatment of capsaicin by itself increased these mRNAs in cultures by 100% to over 200%, as compared to the control group, in a dose-dependent manner (Figure 3A, right panel). The expression of COL1A1 mRNA was highly sensitive to the challenge of capsaicin of having ∼200% increase of control under 10 μM capsaicin. By using antibody against COL1 in western blot, the expressions of COL1 α1 (∼138 kDa) and α2 (∼129 kDa) were both up-regulated by capsaicin in UVB- (Figure 3B left panel) or non-UVB-exposed conditions (Figure 3B right panel). TGF-β1 was a control here.

**FIGURE 4:**
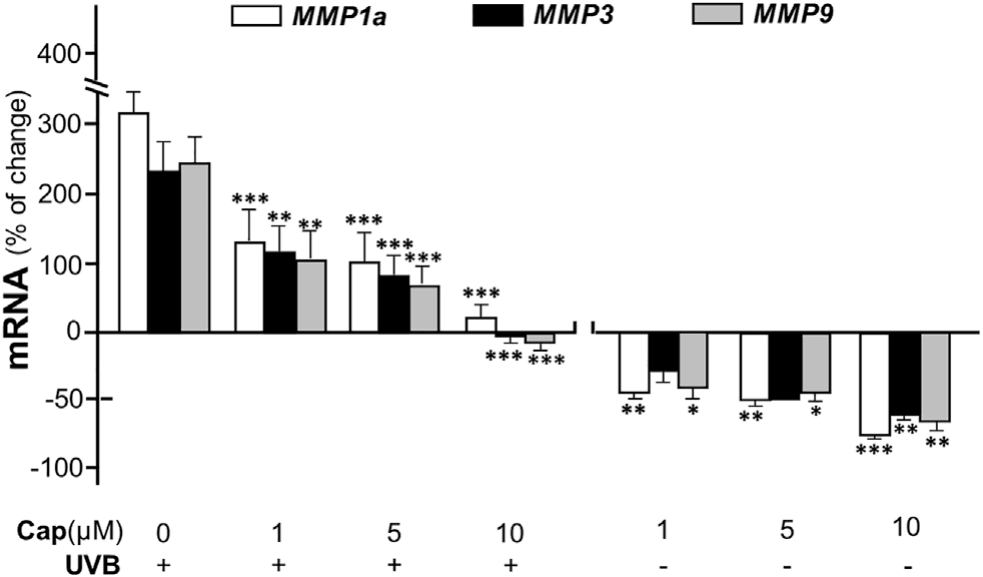
Capsaicin alleviates UVB-induced increase of MMPs in fibroblasts. The dermal fibroblasts were isolated from postnatal day 2 mice and cultured *in vitro*. Cells were incubated with capsaicin (Cap) from 1 to 10 μM with or without UVB irradiation at 300 mW/m^2^ at 300 mW/m^2^ for 5 min. The mRNAs of MMP1a, MMP3, and MMP9 were determined using qRT-PCR. Values are in percentage of change against control, in mean ± SD, *n* = 5, each with triplicate samples. *, *p* < 0.05; **, *p* < 0.01; ***, *p* < 0.001 compared with control (vehicle only).

Matrix metalloproteinases (MMPs) are known as collagenase and the UVB-induced expressions of MMP1, 3, and 9 are considered as the dominant cause of decreased collagen in skin (Kang et al., 2020). In cultured dermal fibroblasts, the transcriptional levels of different MMP isoforms, i.e., MMP1a, MMP3, and MMP9, were increased markedly by few folds under UVB irradiation (Figure 4). The increase in MMP could be accounted for the cause of decreased collagen in fibroblasts. The mRNAs of MMPs, as induced by UVB exposure, were markedly reduced by pre-treatment of capsaicin: the MMP expression was restored to normal level under a treatment of 10 μM capsaicin (Figure 4). Capsaicin alone showed a reduction of MMP in cultured fibroblasts, in a dose-dependent manner (Figure 4 **)**. These results implied that capsaicin increased the collagen protein content through suppressing the collagen degradation as triggered by MMPs.

**FIGURE 5:**
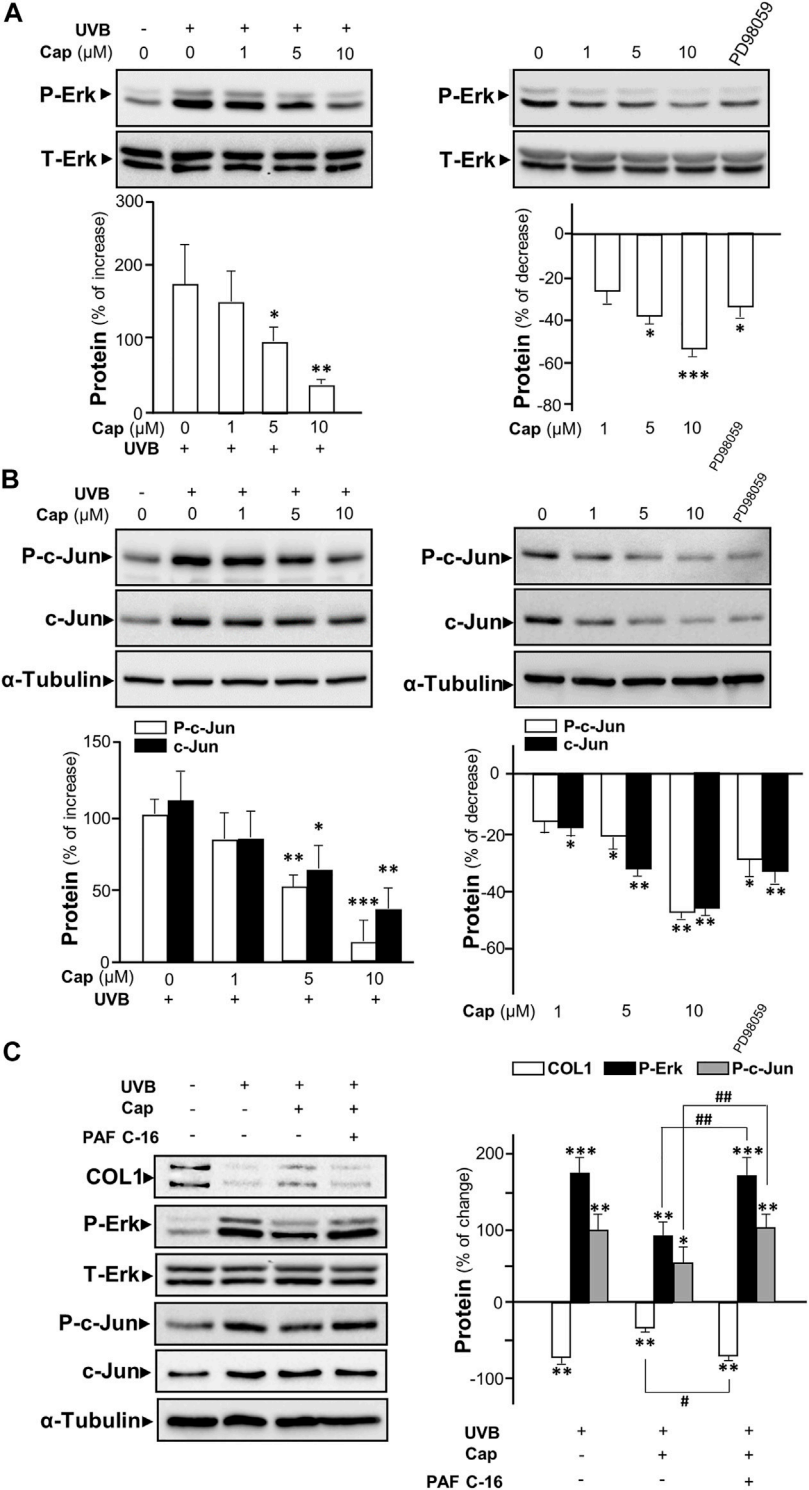
Capsaicin inhibits UVB-induced Erk phosphorylation in cultured fibroblasts. The fibroblasts were incubated with capsaicin (Cap) from 1 to 10 μM with or without UVB irradiation at 300 mW/m^2^ for 5 min. **(A)** The protein lysates were collected for SDS-PAGE and Western blot using antibody against phosphorylated Erk (P-Erk at ∼40 and ∼42 kDa). Antibody against total Erk (T-Erk at ∼40 and ∼42 kDa) was used as a loading control (upper panel). The quantification of the blots was shown (lower panel). **(B)** Antibodies against phosphorylated c-Jun and total c-Jun were applied in Western blot. Antibody against α-tubulin was used as a loading control. PD98059 (10 μM) was used as a positive control here. **(C)** The fibroblasts were treated with capsaicin (1 μM) or PAF C-16 (1 μM) before UVB irradiation. The COL1, P-Erk, T-Erk, P-Jun, and T-Erk were determined (left panel) and qualified (right panel). Values are in percentage of change, or increase, or decrease, against control, in mean ± SD, *n* = 5, each with triplicate samples. *, *p* < 0.05; **, *p* < 0.01; ***, *p* < 0.001 compared with control (vehicle only); #, *p* < 0.05 and ##, *p* < 0.01 compared with corresponding group.

The UV-induced phosphorylated Erk has been reported to increase the expressions of MMP1, 3, and 9 in dermal fibroblasts (Kim et al., 2005; Alge-Priglinger et al., 2009). To determine the role of capsaicin in affecting MMP expression through the Erk phosphorylation, Western blot was performed from cultured fibroblasts being treated with capsaicin with or without UVB irradiation. Exposure of UVB induced Erk phosphorylation (∼40 and ∼42 kDa) by ∼200% (Figure 5A left panel). Capsaicin notably inhibited the UVB-induced phosphorylation of Erk in a dose-dependent manner (Figure 5A, left panel). The Erk phosphorylation was almost back to control level after treatment of 10 μM capsaicin. In the absence of UVB irradiation, the treatment of capsaicin reduced the level of phosphorylated Erk in a dose-dependent manner (Figure 5A, right panel). PD98059, an inhibitor of Erk phosphorylation, was applied here as a positive control. Total level of Erk was not changed in all cases.

The phosphorylated c-Jun is part of AP-1 complexes (Huang et al., 1991), which is known as an activator for the expression of MMP gene (Poulalhon et al., 2006). In cultured fibroblasts, UVB exposure caused an increase of ∼100% of phosphorylated c-Jun, as well as the total c-Jun (Figure 5B left panel). Capsaicin reduced the expression of both phosphorylated and total c-Jun with **(** Figure 5B left panel**)** or without (Figure 5B right panel) UVB irradiation in a dose-dependent manner. In addition, PAF C-16, an activator of MAP kinase inducing Erk phosphorylation, was added to the culture before capsaicin and UVB treatment. PAF C16 reversed the capsaicin-induced increase of collagen after UVB damage. Meanwhile, the inhibition of UV-induced phosphorylations of Erk and c-Jun by capsaicin was dramatically attenuated by the applied PAF C-16 (Figure 5C). Hence, our results indicated that capsaicin increased the collagen content could be through inhibiting Erk/c-Jun-mediated MMP signaling.

### Capsaicin Induces Collagen Not *via* TRPV1

**FIGURE 6:**
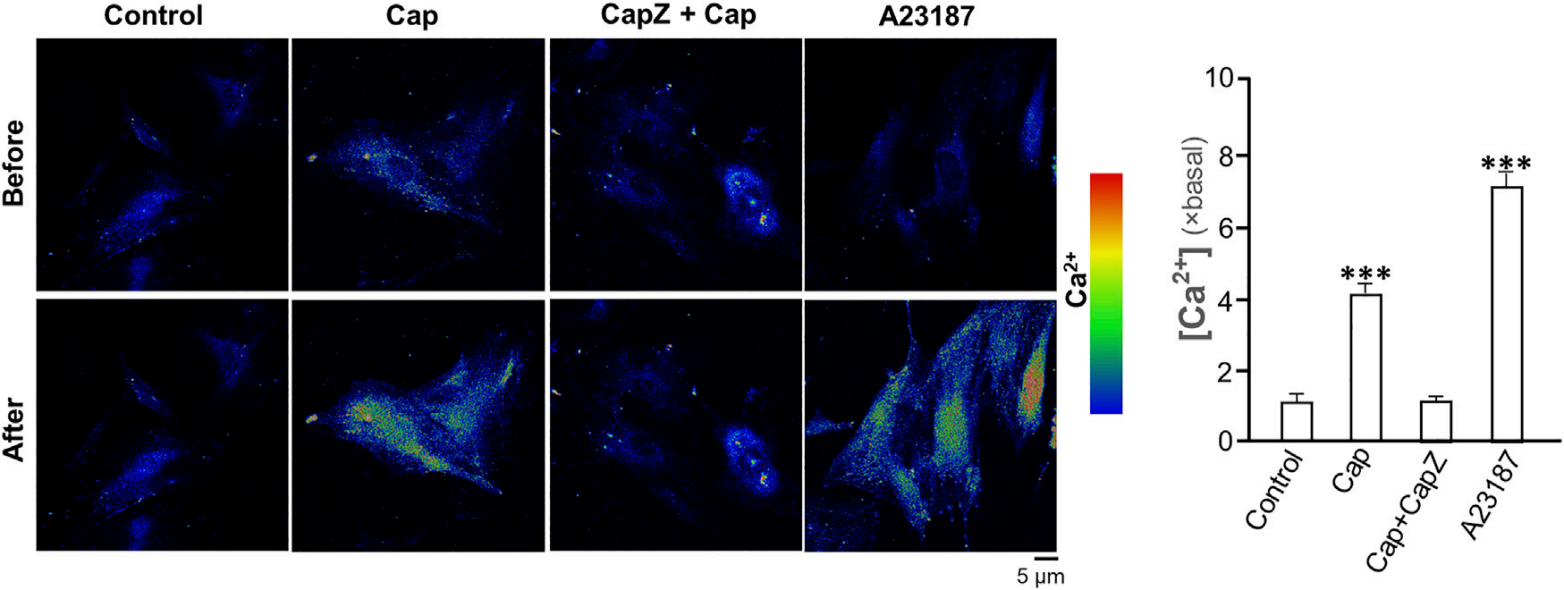
Capsaicin induces Ca^2+^ influx *via* TRPV1 channel in cultured fibroblasts. Cultured fibroblasts were incubated with Ca^2+^ indicator fluo-4 AM (2 μM) and then treated with capsaicin (Cap, 1 μM), capsaicin (1 μM) plus capsazepine (Capz, 10 μM), and the calcium ionophore A23187 (2 μM) for 1 min. The cytosolic Ca^2+^ before and after drug treatment were imaged using confocal microscope. The quantification of the fluorescent signal was shown in right panel. Values are in fold of change (× basal) against control, in mean ± SD, *n* = 5, each with triplicate samples. *, *p* < 0.05; **, *p* < 0.01; ***, *p* < 0.001 compared with control (vehicle only).

**FIGURE 7:**
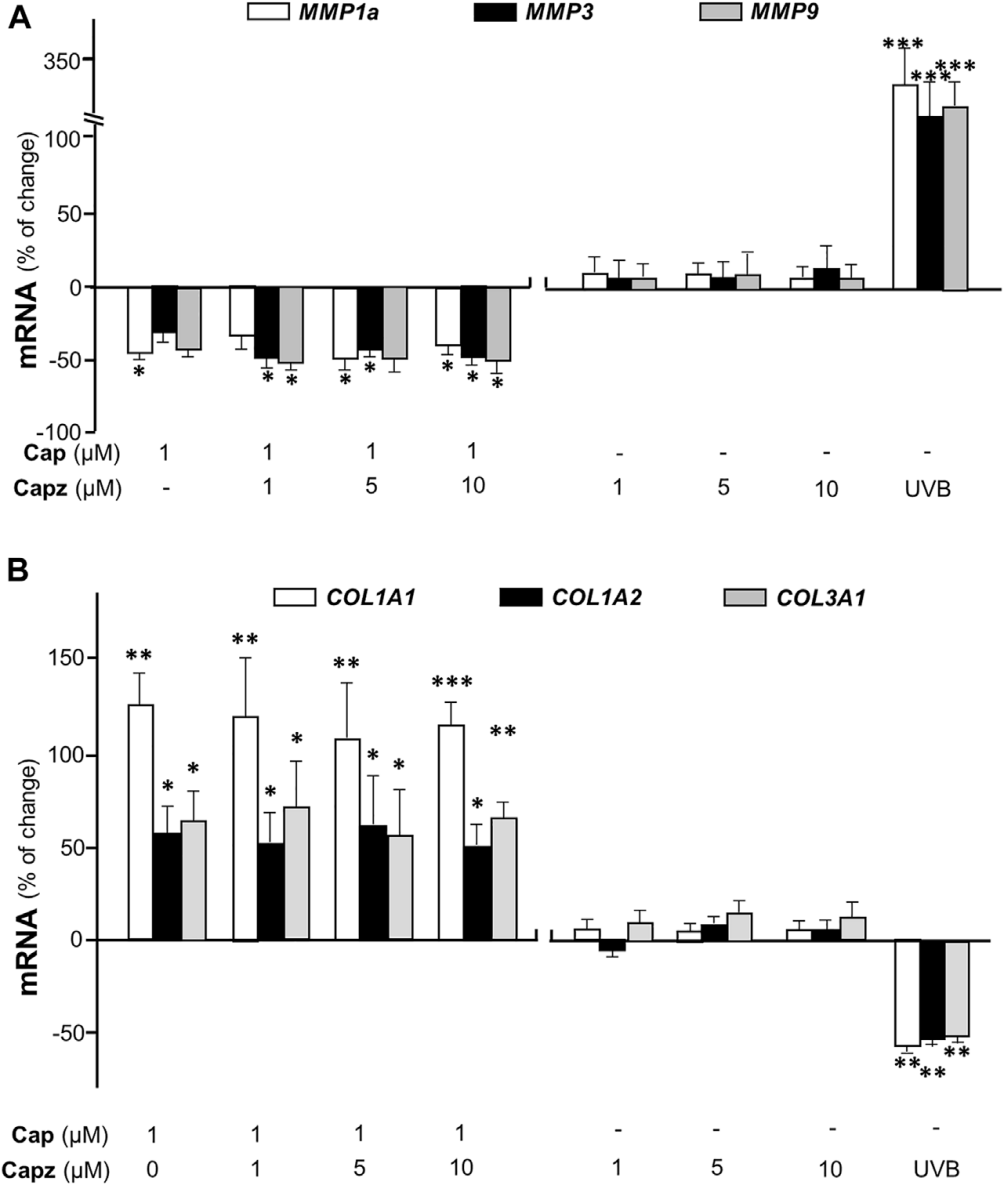
Capsaicin regulates the expression of collagen not through TRPV1 channel. Cultured fibroblasts were treated with capsaicin (1 μM) plus different concentrations of capsazepine (1–10 μM). The mRNAs of **(A)** MMP1a, MMP3, MMP9 **(B)** COL1A1, COL1A2, and COL3A1 were determined using qRT-PCR. UVB irradiation (300 mW/m^2^ for 5 min) was applied as a positive control. Values are in percentage of change against control, in mean ± SD, *n* = 5, each with triplicate samples. *, *p* < 0.05; **, *p* < 0.01; ***, *p* < 0.001 compared with control (vehicle only).

TRPV1 is known as the capsaicin receptor detecting the sensation of heat and pain (Szallasi and Sheta, 2012). Capsaicin activates TRPV1 and changed Ca^2+^ permeability, which results in an intracellular Ca^2+^ influx to cells in triggering multiple cellular responses (Zhang et al., 2020). Our previous work has revealed that capsaicin activates TRPV1 in skin melanocytes to boost PKC activity, resulted in the inhibition on melanin synthesis (Wu et al., 2020). To verify the role of TRPV1 in capsaicin’s effect on skin fibroblasts, we examined the Ca^2+^ influx in capsaicin-treated fibroblasts. The results indicated that capsaicin significantly induced Ca^2+^ influx, which could be blocked fully by pre-treatment of capsazepine, a TRPV1 antagonist (Figure 6). This result was consistent with that in our previous report on melanocyte. A23187, a calcium ionophore, was applied as a positive control here. In contrast, the transcriptional levels of MMPs were not affected by blocking TRPV1 with capsazepine in cultures (Figure 7A). In addition, capsazepine itself from 1 to 10 μM had no effect on the transcriptions of those MMP genes (Figure 7A). In parallel, the expressions of COL1A1, COL1A2, and COL3A1, as induced by capsaicin, were not affected by capsazepine at all (Figure 7B), suggesting that the induction of collagen and MMP by capsaicin should not be mediated by TRPV1.

### Capsaicin Reduces UV-Induced ROS Generation

**FIGURE 8:**
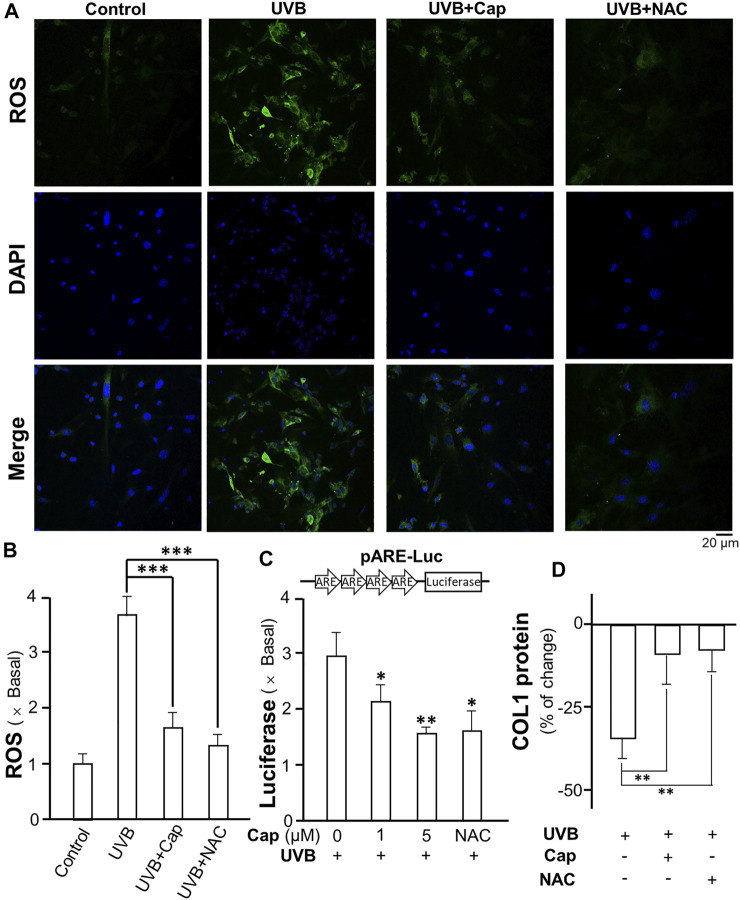
Capsaicin reduces the UVB-induced ROS generation in cultured fibroblasts. **(A)** The cultured fibroblasts were pre-treated with capsaicin (Cap, 1 µM) or N-acetyl-l-cysteine (NAC, 5 mg/ml) for 30 min, followed by exposure to UVB irradiation (300 mW/m^2^ for 5 min). Cells were incubated with DCFH-DA (20 µM) and nucleus were stained with DAPI. Images were captured by confocal microscope. **(B)** The amount of ROS was quantified. **(C)** The culture fibroblasts were transfected with pARE-Luc constructs, as shown, and then treated with capsaicin (Cap, 1 and 5 µM) or NAC (5 mg/ml) with UVB irradiation (300 mW/m^2^ for 5 min). The protein lysates were collected for luciferase assay. **(D)** The protein lysates of cells were collected for ELISA assay to measure COL1 content. Values are in percentage of change, or fold of change (X basal), against control, in mean ± SD, *n* = 5, each with triplicate samples. *, *p* < 0.05; **, *p* < 0.01 compared with control (vehicle only).

Exposure to UVB is known to stimulate ROS generation in skin cells, i.e., keratinocytes and fibroblasts (Marionnet et al., 2010). In dermal fibroblasts, ROS decreased the production of collagen and glucosaminoglycan (Tanaka et al., 1993). The formation of ROS in cultured dermal fibroblasts was measured after UVB irradiation: the intracellular ROS was detected by incubation of the cells with DCFH-DA probe. UVB obviously stimulated ROS generation (green) in cultured fibroblasts (Figure 8A). The pre-treatment of capsaicin before UVB irradiation significantly decreased the amount of ROS to ∼1.5 folds of control; while N-acetyl-l-cysteine (NAC) was used as a positive control to reduce ROS formation (Figure 8A,B). The transcriptional activity of antioxidant response element (ARE) in fibroblasts was determined using a luciferase reporter system, i.e., pARE-Luc. The result was similar with that of ROS generation, in which the treatment of capsaicin dramatically inhibited the transcription of pARE-Luc, as reflected by the luciferase activity in pARE-Luc-transfected fibroblasts (Figure 8C). In addition, the collagen content was determined by using ELISA. The result indicated that capsaicin and NAC could attenuate the UVB-induced decrease on collagen content (Figure 8D), suggesting that ROS could be the cause of capsaicin-mediated collagen/MMP regulation.

## Discussion

Skin aging has become a topic of increasing concern today, particularly due to an increase of aging population. Both intrinsic and extrinsic factors could result in obvious visible sign of aging appearing on skin surface. The intrinsic aging-induced wrinkle, dry skin, and age spot are inevitable and hard to reverse (Mancini et al., 2014). However, the extrinsic aging, triggered by external environmental factors, such as smoking, sun exposure and poor nutrition, could be relieved by chemical or physical approaches. Solar UV irradiation is one of the primary factors in inducing skin aging, especially the facial aging (Friedman, 2005). The UV irradiation influences the majorly outermost and basal layers of skin. For instance, UV irradiation thickens the epidermis through inhibiting degradation of corneocyte desmosomes and impairing differentiation of keratinocytes (Scott et al., 2012). Collagens are extracellular matrix components to support the elasticity of skin (Huertas et al., 2016); however, UV irradiation breaks skin elasticity through influencing the processes of synthesis and degradation of collagens in fibroblasts (Trautinger et al., 1994). Hence, it is necessary to resist the skin damage caused by UV exposure, and pharmaceutical and cosmetic companies are having great interest in developing anti-aging skin products (Kazanci et al., 2017). Here, we provide several lines of evidence to suggest the anti-aging effects of capsaicin, or extract of its parental fruit, chili pepper.

Capsaicin has been used as a topical medicine with analgesic function for years (Watson, 1994). The desensitization of peripheral nociceptors, induced by repeated or high concentrations of capsaicin, is the main cause of pain relief (Winter et al., 1995). Different lines of evidence suggest that capsaicin relives the inflammatory pain through TRPV1 channel (Davis et al., 2000). As an agonist of TRPV1, capsaicin triggers Ca^2+^-induced signaling to influence multiple cellular activities, including anti-melanogenesis, cytokine synthesis, and cell migration (Hwang et al., 2000; Waning et al., 2007; Wu et al., 2020). However, the effect of capsaicin in inflammatory response is rather controversial. Studies have revealed that capsaicin is able to reduce inflammation in macrophages, neurons and skin cells *via* inhibiting the production of cytokines (Colpaert et al., 1983; Desai et al., 2013). In contrast, capsaicin has been proposed to induce neuronal inflammation (Sumikura et al., 2003; Holst et al., 2011). Thus, this is highly possible that the discrepancy of capsaicin in inflammatory responses could be affected by a variety of factors, which needs to be elucidated further.

Our study revealed that capsaicin markedly reduced the UV-induced decrease of collagen in mouse skin, which was proposed to be attributed by the regulation of Erk and c-Jun signaling. However, the upstream signaling, affected by capsaicin in cultured fibroblasts, are still unclear. Our results indicated that capsaicin increased amount of collagen in fibroblasts; but this was not through TRPV1-mediated Ca^2+^ influx. The TRPV1 antagonist capsazepine at 10 µM could completely block the activation of TRPV1 by capsaicin (Kitamura et al., 2018). In line to this notion, capsazepine (10 µM) blocked the capsaicin-induced Ca^2+^ influx, but which showed no effect on collagen content, as demonstrated in the current study. These findings suggest capsaicin could affect the cellular response by different means. Here, capsaicin markedly inhibited the UVB-induced ROS generation and antioxidant responsive element activity in cultured fibroblasts, which therefore supported the antioxidant effect of capsaicin intracellularly. The capsaicin-mediated ROS in fibroblast could account for: 1) phosphorylation of Erk and c-Jun; 2) downregulation of MMPs and collagen transcription; and 3) decrease of MMP-mediated collagen degradation. In line to this notion, the antioxidant property of capsaicin has been reported *in vitro* (Manjunatha and Srinivasan, 2007; Gangabhagirathi and Joshi, 2015). Even though, the mechanism of how capsaicin affects the ROS generation in cultured skin cells is still not well illustrated, which is worth to investigate in further study.

It is interesting to find that capsaicin reduced the phosphorylated Erk in cultured dermal fibroblasts in present study; while an adverse effect was found that capsaicin enhanced the Erk phosphorylation in melanocytes as reported (Wu et al., 2020). In cultured mouse melanocytes, capsaicin induced Ca^2+^ influx through TRPV1, resulting in an increase of protein kinase C (PKC) activity. Afterwards, the increased PKC activity boosted the phosphorylation of Erk to inhibit the expression of melanogenic enzyme tyrosinase, finally led to a decrease on melanin production. The response of capsaicin in melanocytes was fully sensitive to TRPV1 antagonist capsazepine. Here, the capsaicin-reduced Erk phosphorylation in either UVB or non-UVB irradiated fibroblasts was insensitive to capsazepine, and therefore led to the discrepancy of the phosphorylation between melanocyte and fibroblast. The variance of capsaicin’s functions may be due to the distinct intracellular signaling.

## Conclusion

Our study revealed that capsaicin alleviated the loss of collagen in mouse skin after UV irradiation. This distinct function of capsaicin was mediated by its effect on ROS-regulated Erk and c-Jun signaling. Besides, capsaicin inhibited UVB-induced ROS generation and antioxidant responsive element activity in dermal fibroblasts: these responses were not related with TRPV1-mediated Ca^2+^ influx. The upstream signaling, triggered by capsaicin, in collagen synthesis however requires further exploration. Nevertheless, the current findings suggest that capsaicin has a high potential value to be applied in anti-skin aging.

## Data Availability

The raw data supporting the conclusions of this article will be made available by the authors, without undue reservation.

## Supplementary Material

Supplementary Figure S1 The cytotoxicity of capsaicin on keratinocytes and fibroblasts. **(A)** Murine dermal fibroblasts (MDFs) and keratinocytes (MEKs) were isolated from mice at postnatal day 2 and stained with antibodies against collagen type I (COL1) and cytokeratin (KRT) respectively. Nucleus were stained with DAPI. **(B)** MDFs and MEKs were treated with capsaicin from 1 to 100 μM for 24 h. MTT assay was performed to determine the cytotoxicity of capsaicin. Values are in percentage of control, in mean ± SD, *n* = 5, each with triplicate samples. *, *p* < 0.05; **, *p* < 0.01 compared with control (vehicle only).

## Abbreviations

Erk, extracellular signal-regulated kinase; COL, collagen, ROS, Reactive oxygen species; UVB, ultraviolet B; MMP, matrix metalloproteinases; TRPV1, Transient receptor potential cation channel subfamily V member 1
