# Supplementary figures and images for: Capsaicin, a Phytochemical From Chili Pepper, Alleviates the Ultraviolet Irradiation-Induced Decline of Collagen in Dermal Fibroblast via Blocking the Generation of Reactive Oxygen Species
Source: Front Pharmacol. 2022 Mar 14;13:872912. doi: 10.3389/fphar.2022.872912 (PMC8967157; doi:10.3389/fphar.2022.872912)

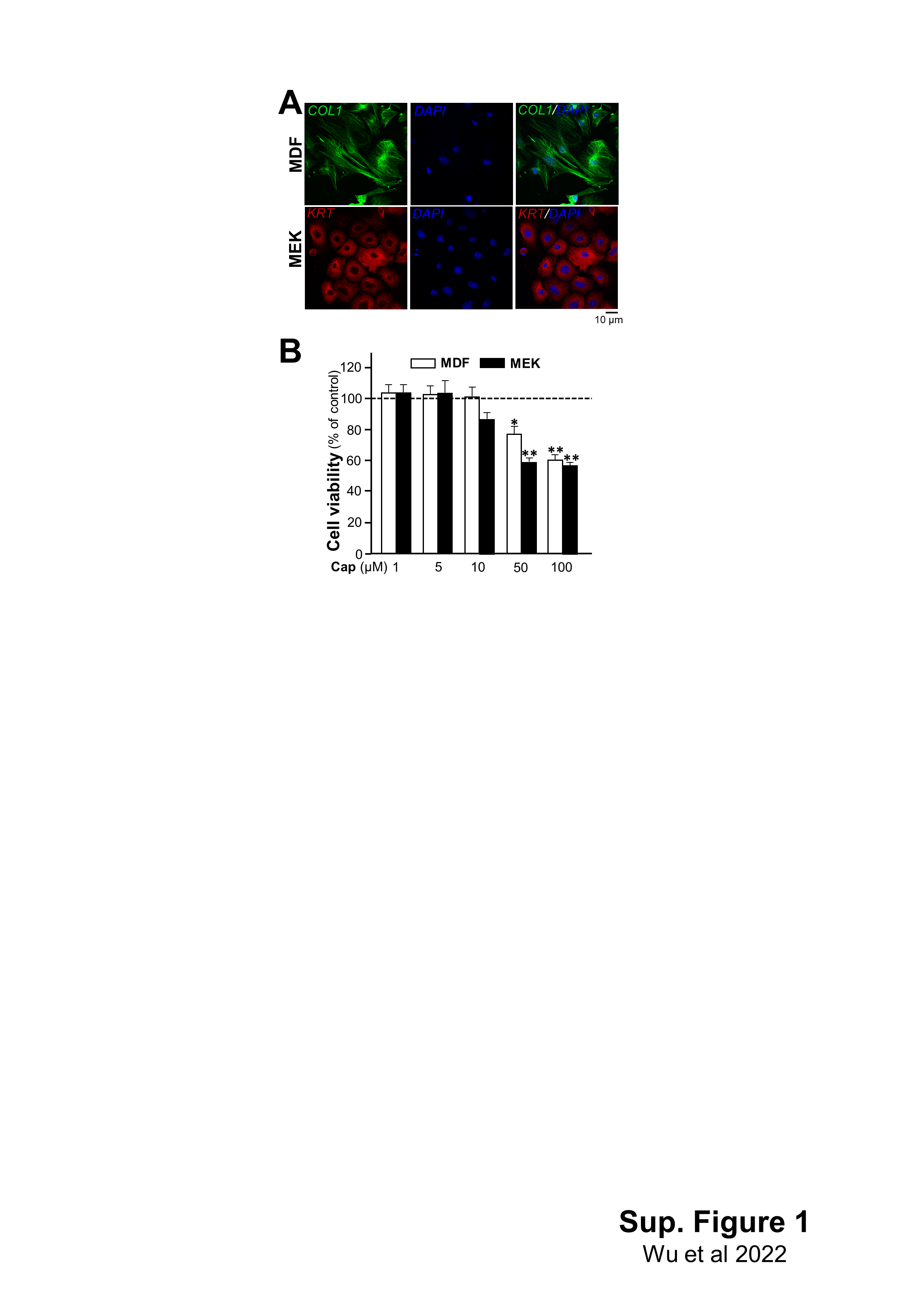

Supplement: Supplementary file 1 [file Image1.tif]
